# *Aphanomyces* *euteiches* causes disease in *Lathyrus* *sativus* with a globally diverse, polygenic resistance landscape

**DOI:** 10.1007/s00122-026-05372-w

**Published:** 2026-09-21

**Authors:** Sara Rodriguez-Mena, Mario González, Ehsan Valiollahi, Carmen Santos, Diego Rubiales, Maria Carlota Vaz Patto

**Affiliations:** 1 Institute for Sustainable Agriculture, CSIC, Córdoba, Spain; 2 Programa de Doctorado de Ingeniería Agraria, Alimentaria, Forestal y del Desarrollo Rural Sostenible, Universidad de Córdoba, Córdoba, Spain; 3 Faculty of Agriculture, Ferdowsi University of Mashhad, Mashhad, Iran; 4 Instituto de Tecnologia Química e Biológica António Xavier, Universidade Nova de Lisboa (ITQB NOVA), Oeiras, Portugal; 5 Present Address: National Institute for Agrarian and Veterinary Research (INIAV), Oeiras, Portugal

## Abstract

**Key message:**

*Aphanomyces euteiches* inoculation of a global* Lathyrus sativus* collection allowed the identification of sources of resistance and associated candidate genes.

**Abstract:**

*Aphanomyces* *euteiches* is a major concern in legume crops with limited efficient methods for its management. Although Aphanomyces root rot has been widely studied in pea and lentil, the effect of this pathogen on grass pea (*Lathyrus* *sativus*) has been poorly explored. This study aimed to characterise the response of a globally diverse grass pea collection to *A.* *euteiches* and to identify genomic regions associated with any potential resistance. A diverse panel of 169 grass pea accessions was inoculated with the *A.* *euteiches* RB84 isolate. Twenty days after inoculation, foliar and root symptoms were evaluated. A wide range of responses was observed within the panel, from susceptibility to partial or even complete resistance. Along with these phenotypic data, an improved acquired 12,974 single-nucleotide polymorphism (SNP) data set was used to perform a genome-wide association study. The association study identified 20 resistance-associated SNPs, across five of the seven grass pea chromosomes, suggesting a polygenic nature. In silico analysis identified seven putative candidate genes associated with resistance involved in vesicle trafficking, signal transduction, nucleotide metabolism, fatty-acid metabolism, cell wall synthesis and ethylene signalling. This study confirms the compatible grass pea × *A.* *euteiches* interaction, identifying for the first time, sources of resistance and proposing resistance-associated loci with co-located candidate genes for future precision breeding purposes.

**Supplementary Information:**

The online version contains supplementary material available at https://doi.org/10.1007/s00122-026-05372-w.

## Introduction

Aphanomyces root rot, caused by the oomycete *Aphanomyces* *euteiches*, is one of the major limitations in pea (*Pisum* *sativum*) and lentil (*Lens* *culinaris*) production worldwide (Wu et al. 2018) and has also been reported on other legumes like some *Lathyrus* species (*Lathyrus* *cicera*, *L.* *hirsutus,* *L.* *latifolius,* *L.* *odoratus* and *L.* *tingitanus*), alfalfa (*Medicago* *sativa*), snap bean (*Phaseolus* *vulgaris*), vetch (*Vicia* *sativa*), faba bean (*V.* *faba*), clovers (*Trifolium* *pratense* and *T.* *repens*), and several weed species (Papavizas and Ayers 1974; Gaulin et al. 2007). This pathogen primarily affects the root system, causing necrosis and discolouration of roots and stems, which leads to yellowing of the leaves and, in severe cases, plant death (Hughes and Grau 2007). It is a genetically diverse pathogen, with different isolates, pathotypes, and field populations identified (Kälin et al. 2022). Traditionally *A.* *euteiches* pea isolates have been classified according to their virulence patterns on differential host sets, distinguishing 11 pathotypes (I to XI) in pea and two races (race 1 and race 2) in alfalfa. (Giles et al. 2022; Moussart et al. 2024). Although *A.* *euteiches* exhibited host specificity, some isolates, such as the pea isolate RB84, could infect several legume species (Leprévost et al. 2026). In cross-infection scenarios, non-specific hosts generally develop root symptoms, whereas aerial ones are absent or delayed (Becking et al. 2022).

Due to their capacity to fix atmospheric nitrogen, legumes play an essential role in sustainable agriculture. An expansion in legume-cultivated areas could also lead to a spread of *A.* *euteiches*, and consequently its impact (Ditzler et al. 2021). Grass pea (*Lathyrus* *sativus*) is a valuable legume crop due to its resistance to drought, flood, salinity, and several pests and diseases, and is considered a model crop for sustainable agriculture (Gonçalves et al. 2024). Although a major constraint on most legume crops, *A.* *euteiches* has not yet been reported as a problem on grass pea, probably due to the decline of this crop cultivation over the past decades in regions where this pathogen is endemic. However, the renewed interest in grass pea cultivation (Gonçalves et al. 2022; Solovieva et al. 2025) highlights the need to investigate the potential effect of Aphanomyces root rot in this crop.

The ability of *A.* *euteiches* to produce long-lasting resting structures (oospores) that can survive in soils over 10 years without a host, combined with its capacity to infect multiple legume species, makes it particularly difficult to control. Some commercial seed treatments, such as Rancona Trio (ipoconazole, carbathiin, and metalaxyl; UPL Agrosolutions, Ontario, Canada) and Zeltera Pulse (ethaboxam, inpyrfluxam, mandestrobin, and metalaxyl; Nufarm, Alberta, Canada), inhibit *A.* *euteiches* infection at the early seedling stage, but they are ineffective when infection occurs at later plant growth stages (Willsey et al. 2021). The incorporation of green manures from Brassicaceae and other soil amendments (e.g., spent lime) has shown efficacy in suppressing Aphanomyces root rot, although their large-scale application is limited (Hossain et al. 2012). Therefore, cultural practices remain the primary management strategy for this pathogen, including long-term crop rotation of at least six to eight years with non-host species, cultivation in well-drained fields, and avoidance of soil compaction (Wikström et al. 2025). Additionally, determining the inoculum potential of the soil prior to sowing is highly recommended in regions where *A.* *euteiches* is endemic, avoiding planting in high infested soils (Chatterton et al. 2023).

Overall, the development of resistant legume varieties represents a key strategy for sustainably managing this disease. Significant research efforts have focused on understanding the genetic basis of plant responses to this pathogen, leading to the identification of sources of resistance and associated genomic regions with the objective of increasing the efficiency of resistance breeding programmes (Bonhomme et al. 2019; Jacquet and Bonhomme 2019; Wu et al. 2021; Leprévost et al. 2023; Rodriguez-Mena et al. 2025). Genetically close legumes susceptible to Aphanomyces root rot (*P.* *sativum*, *V.* *faba*, *L.* *culinaris* and *M.* *truncatula*) have been shown to harbour syntenic QTLs (Leprévost et al. 2026). However, grass pea was not included in previous studies, leaving the presence of syntenic QTLs in this species unexplored. A more comprehensive understanding of the genomic conservation and diversity of resistance loci among co-cultivated legumes could support more rational crop rotation strategies to manage this pathogen with higher efficiency (Mundt 2014). Furthermore, intensifying the cultivation of crops that share similar genetic factors may exert selective pressure on pathogen populations, potentially accelerating adaptation and reducing the durability of host genetic resistance (Papaïx et al. 2011).

As observed in other closely related legumes, grass pea is also expected to exhibit quantitative resistance to Aphanomyces root rot. Although monogenic complete resistance can be highly effective, the mixed reproductive system combining sexual and asexual stages of this pathogen and its high population genetic diversity make quantitative resistance a more durable long-term strategy (Mundt 2014; Kälin et al. 2022). Moreover, pyramiding multiple resistance QTLs that vary in effect across pathogen populations or act at different stages of the pathogen life cycle represents a promising approach to enhance both the resistance and its durability (Pilet-Nayel et al. 2017).

The aim of this study was to evaluate the response of grass pea to *A.* *euteiches* inoculation, clarify the genetic basis of any depicted resistance, and identify associated genetic markers and candidate genes through a genome-wide association study (GWAS) approach. For this purpose, a worldwide diverse collection of 169 grass pea accessions was inoculated with the *A.* *euteiches* RB84 isolate under controlled conditions. Foliar and root symptomatology were assessed 20 days after inoculation. These phenotypic data were then combined with an improved acquisition of a previously generated high-density single-nucleotide polymorphism (SNP) data set to identify SNPs significantly associated with grass pea resistance to *A.* *euteiches* and to propose potential candidate genes.

## Material and methods

### Plant material

The 169 grass pea accessions evaluated belong to the collection described in Sampaio et al. (2021). Among them, 67 accessions had Mediterranean origin, 49 South Asian, 18 East European, 15 Sub-Saharan, 12 North Asian, 2 South American, 2 North American and 4 with unknown origin. Seeds also had differences in seed colour, with 96 of them dark and 73 light (based on visual score), and in seed size, with 45 of them large (100 seed weight ≥ 18 g) and 124 small (100 seed weight < 18 g).

For the experiments, seeds were scarified and surface-sterilised by immersion in a 10% of sodium hypochlorite solution for 10 min followed by rinse with water for 10 min. After sterilisation, seeds were placed in Petri dishes wrapped with moist paper. A cold shock was applied by incubating the plates at 4 ºC in darkness for 24 h. Plates were then transferred to a growth chamber and kept in darkness at 23 ± 2 ºC for 3 days. Germinated seeds were sown in 200 mL plastic pots (6 × 6 × 10 cm) filled with autoclaved perlite. Pots were placed in plastic trays (8 × 6 pots per tray) and maintained under controlled conditions (chamber at the Institute of Sustainable Agriculture 24 ± 2 ºC, 65 RH, 12:12 h D:N with 150 µmol·m^−2^·s^2^). Plants were irrigated every 3−4 days with Hoagland nutrient solution (Hoagland and Arnon 1938).

The accessions were evaluated using a completely randomised block design across three temporally independent rounds. Each round included four biological replicates per accession, randomly assigned to individual trays. The susceptible pea (*P.* *sativum*) cultivar “Messire” was included as a positive control in each tray, following the protocol described by Rodriguez-Mena et al. (2024).

### Inoculation and disease assessment

Zoospores of *A.* *euteiches* were produced as described by Rodriguez-Mena et al. (2024). Zoospore concentration was adjusted to 1000 zoospores/mL using a Fuchs-Rosenthal hemocytometer. Ten-day-old plants were inoculated by applying 5 mL of the suspension at the base of each stem.

**Fig. 1:**
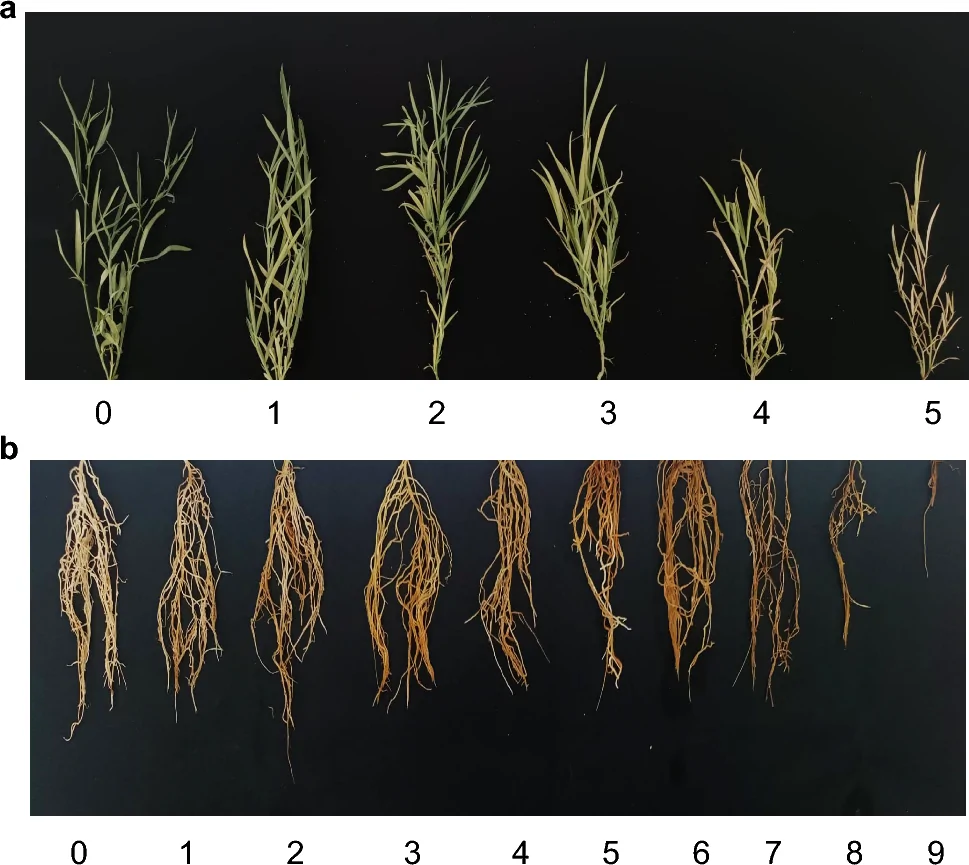
Scales of evaluation for grass pea symptoms after the inoculation with *Aphanomyces* *euteiches*. **a** Foliar symptom scale from 0 (healthy) to 5 (dead). **b** Root rot scale from 0 (healthy) to 9 (dead)

Similar as it is performed in pea, foliar and root symptoms were assessed 20 days post-inoculation (Rodriguez-Mena et al. 2025). Foliar symptoms (FS) were rated on a scale from 0 (healthy plant) to 5 (dead plant) (Fig. 1a), while root symptoms (RS) were evaluated using a scale from 0 (healthy plant) to 9 (dead plant) (Fig. 1b).

### Phenotypic data analysis

Statistical analyses were performed using R software version 4.4.2 (R Core Team 2024) with “ggplot2” package for data visualisation (Wickham 2016). First, outliers were detected by the interquartile range method and removed in the required cases (Estaghvirou et al. 2014; Tripodi 2022). The effect of the experimental components (round, repetition and genotype) on the evaluated symptoms was assessed with a Kruskal–Wallis test due to their ordinal nature. Then, the correlation between FS and RS was calculated using the *stat_poly_eq* function from “ggmisc” package (Aphalo et al. 2024). Effects of seed characteristics, including geographical origin, colour and size, on both symptoms were evaluated with Kruskal–Wallis test followed by a Dunn test in the required cases (*p*-value < 0.05) (functions *Kruskal.test* and *DunnTest*, Bonferroni method from the “stats” package (R Core Team 2024)).

Foliar and root rot symptoms were analysed as ordinal variables using a cumulative link mixed model with logit as link function (*clmm* function from the “ordinal” package (Christensen 2019)) (Leprévost et al. 2026). Phenotypic data were adjusted to the completely random model: FS/RS = *Genotype* + *Round* + *Round:Genotype* + *Round:Repetition* where *Genotype* represents the 169 grass pea evaluated accessions, *Round* the 3 temporally separate experiments, *Genotype:Round* the interaction between round and genotype components and *Round:Repetition* the interaction between round and the 4 biological replicates contained in each round according to the performed block design. The variance components of this model were used to calculate broad-sense heritability (H^2^) of both symptoms using the formula: ${H}^{2}={\sigma}_{G}^{2}/[{\sigma}_{G}^{2}+{(\sigma }_{E}^{2}/r)]$ where *σ*^2^_G_ is the genetic variance, *σ*^2^_*E*_ the residual variance and *r* the number of temporal rounds performed (Nakagawa and Schielzeth 2010; Leprévost et al. 2026). The same model but, with genotype as fix term, was used to obtain best linear unbiased estimates (BLUEs) (*emmeas* function of the “emmeas” package (Lenth 2025)).

### SNP data acquisition improvement

DNA from each grass pea accession was previously extracted from young leaves and genotyped using two high-throughput genotyping‑by‑sequencing platforms, DArTseq^™^ genotyping (Kilian et al. 2012) and genotype‑by‑sequencing (GBS) performed by BGI (Beijing Genomic Institute, Beijing, China) (Sampaio et al. 2021). In the present study, to improve SNP data acquisition, we took advantage of the recently published grass pea reference genome (accession GCA_963859935.3) (Vigouroux et al. 2024) by calling additional SNPs from the GBS data and by mapping the DArTseq-derived markers onto the genome assembly.

For GBS data, variants were identified using GBS-SNP-CROP v.4.1 (Melo et al. 2016). Clean, demultiplexed reads were mapped to the seven primary chromosomes of the *L.* *sativus* reference genome using the GBS-SNP-CROP-5.pl script, which implements BWA-MEM (Li 2013) and SAMtools (Li et al. 2009) to generate standard alignment files. Variant and genotype calling were then performed using the GBS-SNP-CROP-7.pl script. The resulting SNP genotype matrix was formatted for compatibility with TasselGUI (Glaubitz et al. 2014) for subsequent analysis. For DArTseq markers, the genomic flanking sequences were aligned to the *L.* *sativus* reference genome using the BLASTn tool (e-value < 1 × 10^–5^) implemented in OmicsBox v2.0, and markers with no significant hit or with multiple hits showing identical alignment probability (bit score, percentage similarity and e-value) to different genomic regions were considered unmapped and excluded from further analyses.

Marker quality filtering was performed by removing those with a minor allele frequency (MAF) below 0.05 or with missing data/heterozygosity greater than 0.25, and by removing redundant markers shared between DArTseq and GBS data sets.

As described in Sampaio et al. (2021), a subset of markers was selected by considering 0.1 Mpb interval between markers within each chromosome to be used in the principal components calculation to determine population structure. Linkage disequilibrium (LD) was estimated for all quality-filtered markers as the squared coefficient of correlation between marker pairs (r^2^), after correction for the population structure using the principal components scores from Eigenanalysis. These analyses were performed in Genstat® software, 24th edition (VSN International, 2024).

### Association mapping analysis

BLUEs from FS and RS were used along with the high-quality-filtered SNP markers for association mapping. A single-trait genome-wide association study was performed using GAPIT 3.0 (Wang and Zhang 2021), testing four models: single locus mixed linear model (MLM), multiple locus mixed linear model (MMLM), multi-locus Bayesian information and linkage disequilibrium iteratively nested keyway (BLINK) and fixed and random model circulating probability unification (FarmCPU). The optimal number of principal components (PCs) was determined using the Bayesian Information criterion (BIC)-based model selection approach. Kinship matrix (VanRaden method) was also included to account for relatedness among individuals (Astle and Balding 2009; Zhang 2023). To evaluate the genomic inflation or deflation, lambda (*λ*) of each model was calculated (*estlambda2* function of the “QQperm” package (Wang 2016)) and Quantile–Quantile plots performed for each model and trait. Models with *λ* below 0.9 or higher to 1.2 were not considered.

Significantly associated markers for each model were identified using false discovery rate (FDR) correction with an adjusted *p* value threshold of 0.05 (Chen et al. 2021). Unlike the more conservative Bonferroni correction, FDR provides a less restrictive alternative that increases the likelihood of detecting meaningful associations that might otherwise be overlooked (Storey and Tibshirani 2003; Osuna‐Caballero et al. 2024). *P*-value adjustment was performed using the *p.adjust* function from the “stat” package (R Core Team 2024).

Phenotypic variance explained (PVE) was calculated from the significant associated markers using the formula: $PVE = V_{QTL} / V_{{{\mathrm{phenotype}}}}$ in which V_*QTL*_ represent the variance of the marker calculated as 2·MAF·(1 − MAF)·effect^2^ and V_phenotype_ the genotypic variance of the adjustment model (Resende et al. 2017).

To visualise the results, Manhattan plots were generated displaying the -log_10_ (*p*-value) for each marker along the seven *L.* *sativus* chromosomes highlighting the markers with significant association. Additionally, MG2V version 2.1 was used to represent significantly associated markers distribution across the seven grass pea chromosomes (Chao et al. 2021).

### Partially resistant major allele frequency

Based on their foliar symptom and root rot symptoms average values, the 169 grass pea evaluated accessions were classified into three resistance groups. For FS, the groups were defined as resistant (FS ≤ 1), moderately susceptible (1 < FS ≤ 3) and susceptible (3 < FS ≤ 5). For RS, the thresholds were resistant (RS ≤ 5), moderately susceptible (5 < RS ≤ 7) and susceptible (7 < RS ≤ 9).

For each significantly associated marker, the number of reference, alternative and heterozygotes or missing values was counted within each group. The most frequent allele in resistant group was designated as “partially resistant major allele”. Its frequency was then calculated for each group by dividing the number of partially resistant major allele by the total number of genotypes in that group.

### Candidate genes

A tentative list of putative candidate genes involved in the response was established by exploring the genomic regions surrounding significantly associated SNPs. Based on previously calculated recombination, nearby markers were checked and considered linked when r^2^ > 0.2. If linked markers were present, the search window encompassed the genomic interval between them. In the absence of linked markers, the candidate region was limited to the position of the significant SNP itself (Leitão et al. 2023).

Gene annotation within these regions was manually performed using NCBI genome browser (https://www.ncbi.nlm.nih.gov/datasets/genome/GCA_963859935.3/) (Vigouroux et al. 2024). When possible, corresponding protein FASTA sequences of putative candidate genes were retrieved and subsequently used for functional annotation via Mercator4 v2.0 (https://mapman.gabipd.org/app/mercator) (Schwacke et al. 2019).

## Results

### Grass pea responses to *Aphanomyces euteiches*

**Fig. 2:**
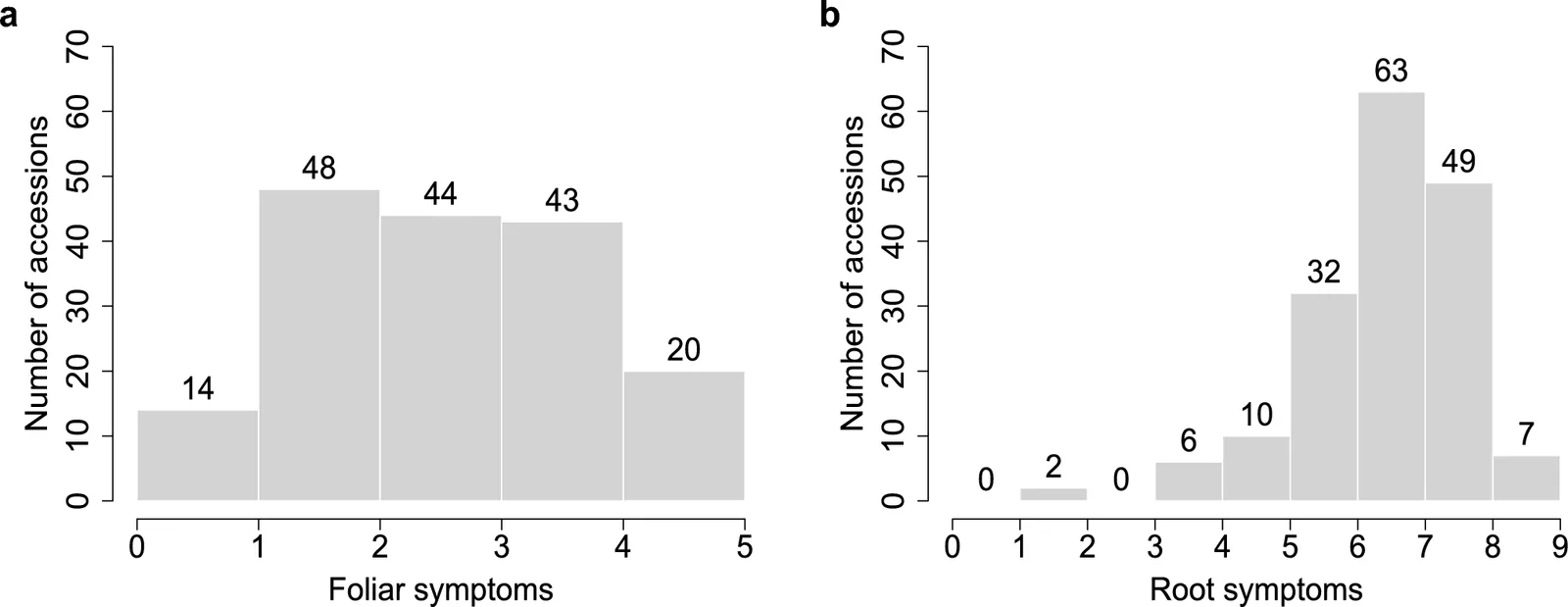
Frequency distribution of average foliar and root symptoms in the 169 grass pea evaluated accessions 20 days after inoculation with *Aphanomyces* *euteiches* under controlled conditions. **a** Foliar symptoms (FS) distribution in a 0 (healthy) to 5 (dead) scale. **b** Root rot symptoms (RS) distribution in a 0 (healthy) to 9 (dead) scale. Means were performed using the evaluations of four biological replicates across the three temporal repetitions (12 plants per accession)

The evaluated grass pea collection presented a wide range in foliar and root symptomatology. FS average values ranged from 0.08 to 4.50 and RS from 1.64 to 8.73 (Table S1). Most of the evaluated accessions presented average FS and RS superior to 1 and 5, respectively, to the tested isolate. Still, 12 resistant accessions with average FS < 1 and RS < 5 were identified, two of them being highly resistant (FS < 0.5 and RS < 2) (Fig. 2). Average FS and RS values of 4.59 and 8.48 observed in the susceptible pea control “Messire” confirmed the adequacy of the inoculation.

Genotype differences were mainly responsible for foliar and root symptomatology variance, with a higher broad-sense heritability associated with RS (Table 1).

**Table 1:** Broad-sense heritability (H^2^), variance components and error for foliar and root symptoms 20 days after the inoculation with *Aphanomyces* *euteiches* in the 169 grass pea evaluated accessions under controlled conditions

		Variance components				
Symptom	Heritability (*H*^2^)	*σ*^2^_Accession_	*σ*^2^_Round_	*σ*^2^_Round:Genotype_	*σ*^2^_Round:Repetition_	Error
FS	0.84	5.17	0.71	1.25	0.12	3.29
RS	0.88	3.79	0.06	0.66	0.05	3.29

**Fig. 3:**
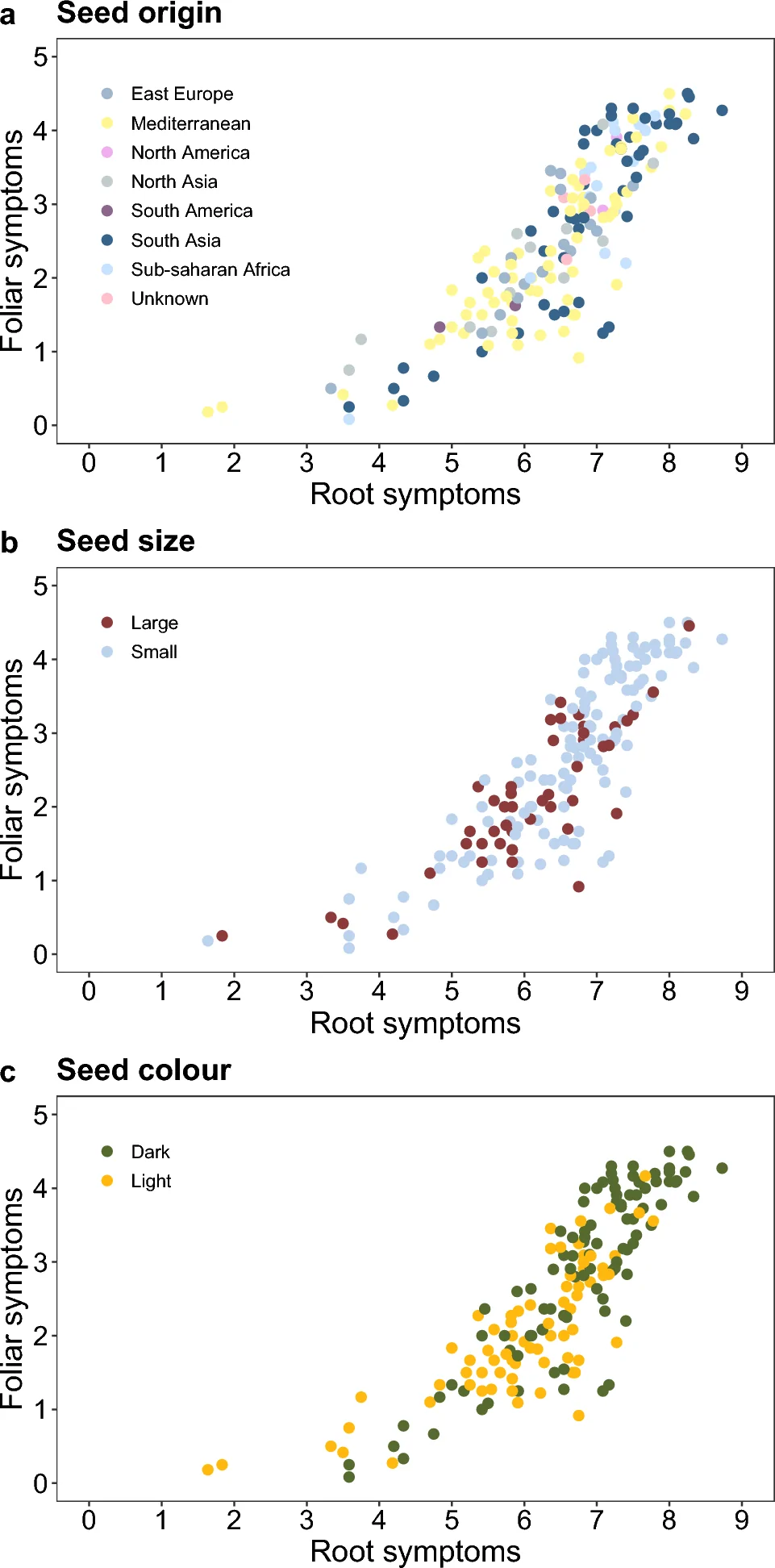
Average foliar and root symptoms of the 169 grass pea evaluated accessions under controlled conditions 20 days after *Aphanomyces* *euteiches* inoculation*.* Means were calculated using the evaluations of four biological replicates across the three temporal rounds (12 plants per accession). Accessions are coloured by seed geographical origin (**a**), size (**b**), and colour (**c**). Linear regression: y = − 2.68 + 0.81 x (R^2^ = 0.71, *p*-value = 9.73 × 10^–47^)

A moderate correlation was observed between FS and RS (*R*^2 ^= 0.71, *p*-value < 0.05) (Fig. 3 and Fig. S1). Significant differences in both symptoms were observed based on seed characteristics (Kruskal–Wallis and Dunn test *p*value < 0.05). Regarding geographical origin, Mediterranean accessions presented significantly lower RS than South Asian ones, with non-significant differences among the rest of the origin groups. With respect to seed size and colour, accessions with large and light seeds displayed lower levels of disease symptoms (Fig. 3 and Table S2).

### Genotyping data improvement and marker positions

**Fig. 4:**
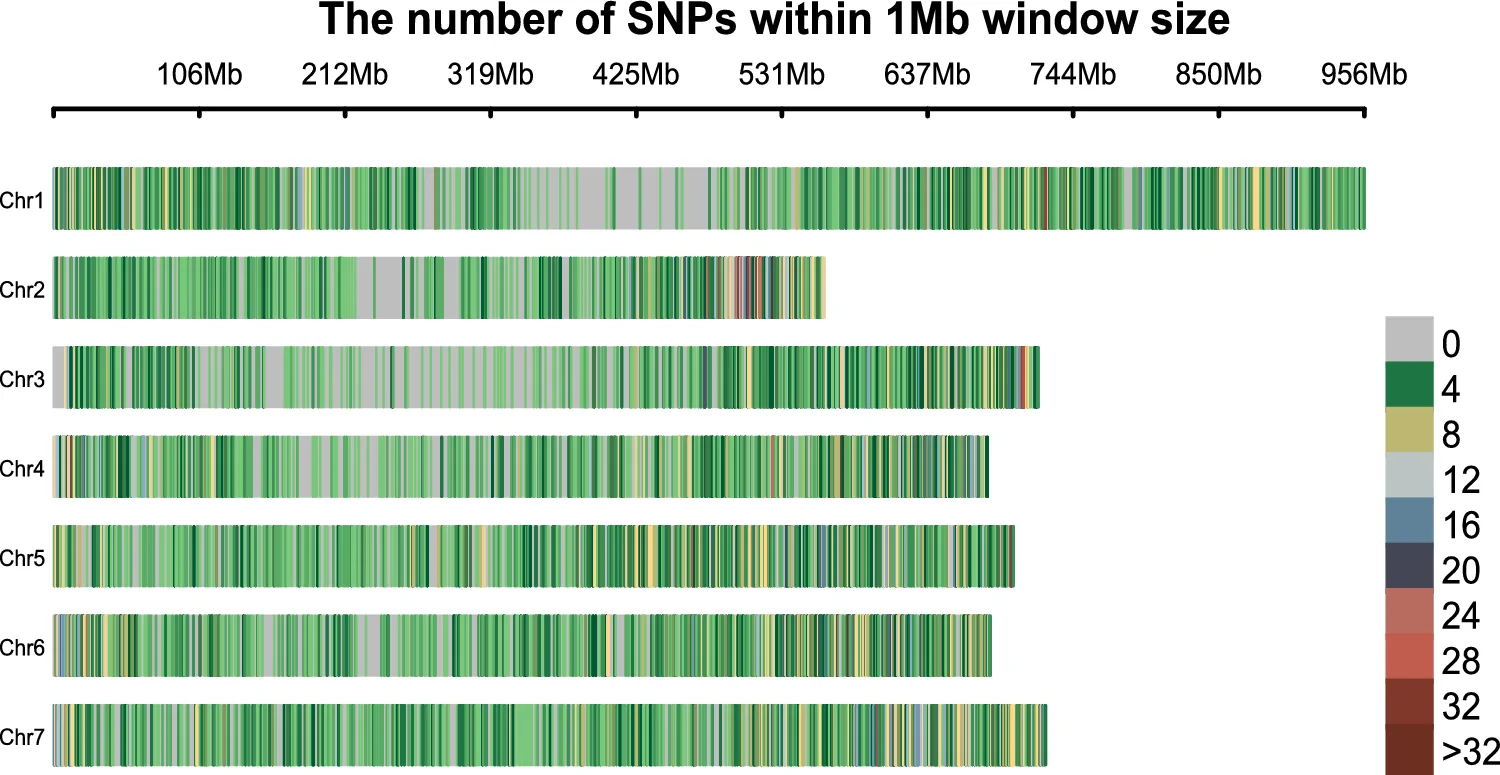
Physical distribution and density of the 12,974 quality-filtered SNP markers, from a combination of DarTseq™ and GBS, across the seven grass pea chromosomes

The DarTseq™ and GBS genotyping combined resulted in 26,876 SNPs, of which 5651 were high-quality (Sampaio et al. 2021). After the improvement of the original SNPs scoring by BGI and DArTseq using the reference grass pea genome (Vigouroux et al. 2024), a total of 44,383 SNPs were detected of which 12,974 SNPs (29.23%) passed the quality control criteria. The quality-filtered SNP markers provided a comprehensive coverage of the seven grass pea chromosomes and were used for association mapping analysis (Fig. 4). SNP marker list and the marker scores per accession are available in Table S3. The subset of markers used to calculate the principal component is listed in Table S4.

### Genetic regions associated to grass pea resistance against *Aphanomyces euteiches*

**Fig. 5:**
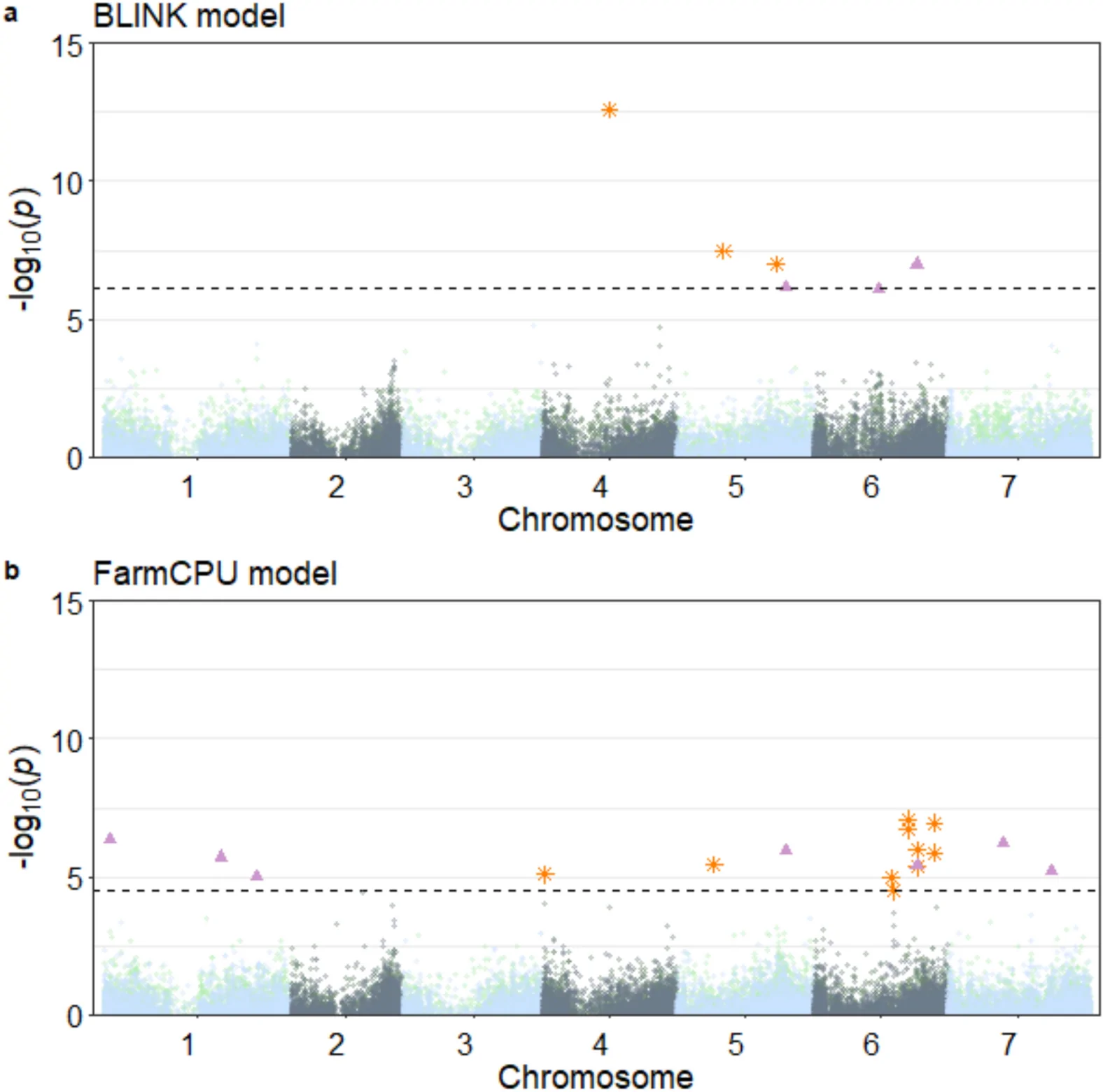
Manhattan plots depicting the -log_10_(*p*-value) versus chromosomal position of quality-filtered 12,974 single-nucleotide polymorphism (SNP) markers associated with foliar (green) and root (blue) symptoms of a 169 accessions grass pea collection 20 days after *Aphanomyces* *euteiches* inoculation under controlled conditions. **a** Markers detected using BLINK model and **b** using FarmCPU model. Dashed horizontal line indicates false discovery rate significance threshold. Orange asterisks and purple triangles represent significant markers associated with foliar and root rot symptoms, respectively

To detect significant markers associated with grass pea resistance against *A.* *euteiches*, a GWAS was conducted testing 4 different models: MLM, MLMM, BLINK, and FarmCPU. Although all the genomic inflation factor (λ) and Q-Q plots revealed that the population structure was efficiently controlled, only BLINK and FarmCPU detected significantly associated markers (Fig. S2). A total of 20 significant markers were identified, two of them common between BLINK and FarmCPU. FS had 13 significantly associated SNPs and RS eight (Fig. 5).

FarmCPU detected a higher number of significantly associated markers than BLINK (Table 2). Sixteen significant associated SNPs were identified by FarmCPU, 9 of them linked with FS, 6 with RS and a common one between both phenotypic traits. On the other hand, BLINK detected 6 significant associated markers, 3 associated with FS and 3 with RS. The 20 significantly associated markers were distributed along 5 of the seven grass pea chromosomes, with none of them located in chromosomes 2 or 3 (Fig. S3). Markers in chromosomes 1 and 7 were only detected with FarmCPU, while chromosomes 4, 5, and 6 contained markers detected with both models. The markers SNP032149 and SNP007517, in chromosomes 5 and 6, respectively, were significantly associated with RS in both models, and SNP007517 was also associated with FS using FarmCPU (Fig. 5 and Table 2). The PVE of the significant markers detected with BLINK ranged between 4.22 to 11.62% and with FarmCPU markers from 0.13 to 9.58%. (Table 2).

**Table 2:** List of significant associated single nucleotide polymorphism (SNP) (adjusted *p*-value < 0.05) with foliar (FS) or root rot (RS) symptoms 20 days after *Aphanomyces euteiches* inoculation in the 169 grass pea evaluated accessions under controlled conditions. Symptoms, marker code, chromosome, position, effect, minor allele frequency (MAF), adjusted *p*-value by the false discovery rate method, percentage of the phenotypic variance explained (PVE), and the model which detected the marker are shown

Symptoms	SNP	Chr	Pos (bp)	Effect^1^	MAF	Adjusted*p*-value	PVE (%)	Model
FS	SNP024285	4	12,829,152	0.75	0.28	1.33×10^–2^	4.34	FarmCPU
	SNP004208	4	339,809,542	1.51	0.16	3.26 × 10^–9^	11.62	BLINK
	SNP005290	5	193,375,403	0.87	0.10	7.56 × 10^–3^	2.72	FarmCPU
	SNP005347	5	241,052,867	1.20	0.12	2.24 × 10^–4^	5.77	BLINK
	SNP031472	5	508,589,669	0.96	0.24	4.50 × 10^–4^	6.48	BLINK
	SNP035346	6	395,296,031	- 0.60	0.50	1.61 × 10^–2^	3.49	FarmCPU
	SNP035419	6	408,765,593	0.55	0.25	4.46 × 10^–2^	2.19	FarmCPU
	SNP035948	6	478,139,587	- 0.38	0.17	8.72 × 10^–4^	0.80	FarmCPU
	SNP035962	6	478,197,292	1.35	0.17	7.29 × 10^–4^	9.58	FarmCPU
	SNP007517^♦^	6	525,489,889	0.83	0.26	3.52 × 10^–3^	5.08	FarmCPU
	SNP007520	6	525,490,364	0.13	0.27	8.30 × 10^–3^	0.13	FarmCPU
	SNP038080	6	616,109,525	0.24	0.26	7.29 × 10^–4^	0.42	FarmCPU
	SNP038082	6	616,109,553	0.87	0.31	3.52 × 10^–3^	6.22	FarmCPU
RS	SNP011771	1	32,448,627	- 0.82	0.30	4.22 × 10^–3^	7.46	FarmCPU
	SNP000794	1	595,681,698	0.73	0.13	6.49 × 10^–3^	3.19	FarmCPU
	SNP001216	1	779,876,981	0.51	0.39	2.00× 10^–2^	3.25	FarmCPU
	SNP032149*	5	561,173,187	- 0.73	0.26	3.50 × 10^–3^	5.51	BLINK
	SNP032149*	5	561,173,187	- 0.67	0.26	4.81 × 10^–3^	4.55	FarmCPU
	SNP035033	6	328,033,297	- 0.72	0.19	3.50 × 10^–3^	4.22	BLINK
	SNP007517*	6	525,489,889	0.82	0.26	1.35 × 10^–3^	6.75	BLINK
	SNP007517*^♦^	6	525,489,889	0.70	0.26	1.05 × 10^–2^	4.91	FarmCPU
	SNP040001	7	282,585,828	- 0.81	0.31	4.22 × 10^–3^	7.42	FarmCPU
	SNP041226	7	531,394,642	1.05	0.17	1.42 × 10^–2^	8.30	FarmCPU

^1^Effect of the minor allele

^*^Common markers between models

^♦^Common markers between both symptoms

### Marker allele frequency based on symptomatology

Based on the average values of foliar and root symptoms, the 169 grass pea evaluated accessions were classified into 3 categories: resistant, moderately susceptible, and susceptible. For FS, 14 accessions were classified as resistant (FS ≤ 1), 92 as moderately susceptible (1 < FS ≤ 3), and 63 as susceptible (3 < FS ≤ 5). For RS, 18 accessions were classified as resistant (RS ≤ 5), 95 as moderately susceptible (5 < RS ≤ 7), and 56 as susceptible (7 < RS ≤ 9).

**Fig. 6:**
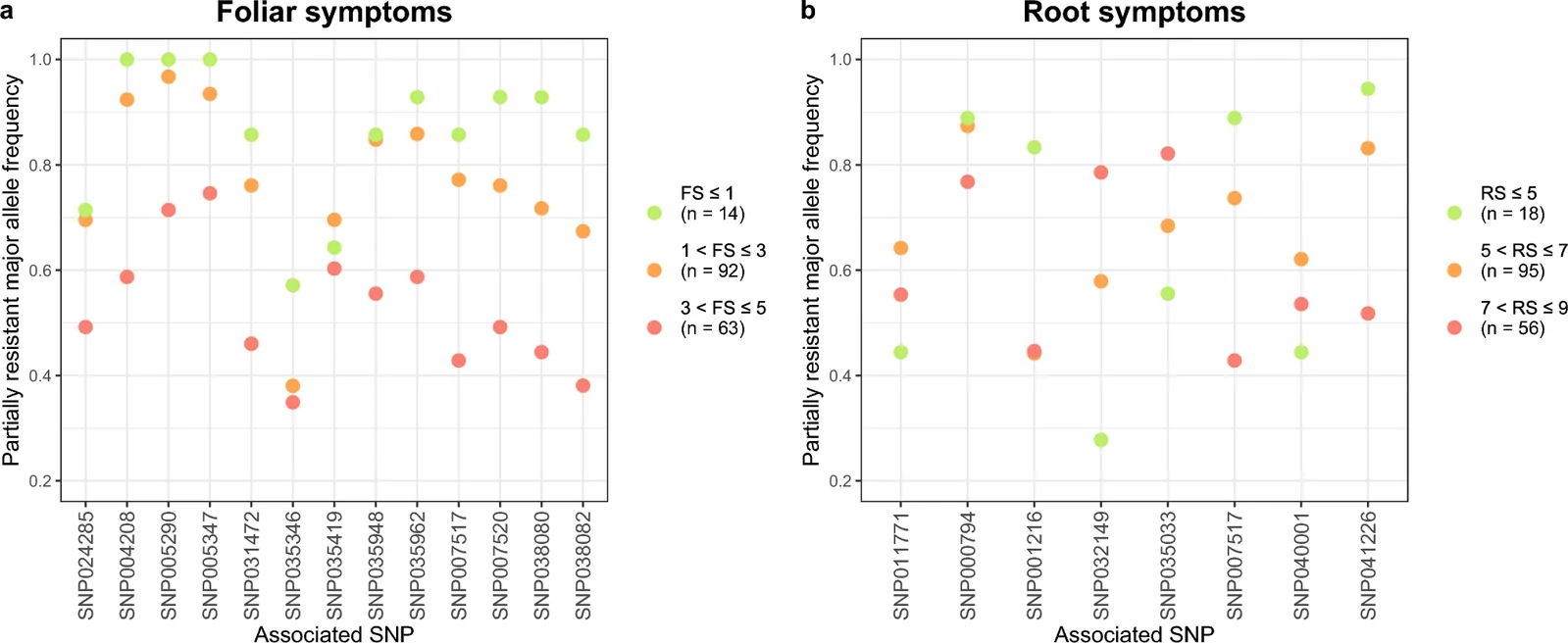
Frequency of the partially resistant major allele (predominant in the resistant group) of the significant associated single-nucleotide polymorphism (SNP) markers linked with foliar (FS) (**a**) and root (RS) symptoms (**b**). The 169 grass pea evaluated accessions were classified into three groups based on their average foliar and root symptomatology 20 days after *Aphanomyces* *euteiches* inoculation under controlled conditions

Differences in the partially resistant major allele frequency (most frequent in resistant group) were observed between resistant groups across the significantly associated markers, with the clearest differences occurring in FS-associated markers. For FS-associated markers, the frequency of the partially resistant major allele was significantly higher in the resistant group compared to the susceptible group in all cases except SNP035419. In contrast, for RS associated markers, this exception occurred with SNP011771, SNP032149, SNP035033 and SNP040001. In these cases, the lower frequency in the resistant groups was justified by a high number of heterozygotes or missing values among the resistant accessions (Fig. 6 and Tables S5 and S6).

### In silico identification of candidate genes

Using an LD decay threshold of r^2 ^= 0.2, SNP035033, SNP035419, and SNP038082 were found to be in LD with SNP035035, SNP035415, and SNP038086, respectively. For these SNP pairs, the candidate gene search window was extended to span the region between the linked markers. In the rest of the cases, the search window was restricted to the position of the associated SNP (Table S7).

Seven of the twenty significantly associated markers were located within annotated *L.* *sativus* genes. Of them, three were associated with FS, three with RS and one was common to both phenotypic traits. Most of the candidate genes were located on chromosome 6 (three), while the remaining four were distributed across chromosomes 1, 4, 5 and 7, with one candidate gene identified on each of these chromosomes.

Diverse biological functions were associated with the candidate genes. In chromosome 1, *g3062.t1* encoded a wax ester synthase. Chromosome 4 harboured *g14004.t1* which coded a component of the exocyst complex. Chromosome 5 contained *g21044.t1* gene that encoded a protein implicated in fatty-acid metabolism. Chromosome 6 contained two protein kinase genes (*g24166.t1* and *g24512.t1)* and an ethylene receptor gene (*g24922.t1*). Finally, *g29459.t1*, a callose synthase coding gene, was located on chromosome 7 (Table 3).

**Table 3:** Grass pea genes containing significantly associated markers with foliar (FS) or root symptoms (RS) 20 days after *Aphanomyces* *euteiches* inoculation, including gene and protein IDs and functional annotations according to MapMan/Mercator, Prot-scriber and Swissprot

Symptoms	SNP	Chr	Pos (bp)	Gen ID	Protein ID	Mercator	Prot-scriber	Swissprot
FS	SNP024285	4	12,829,152	*g14004.t1*	CAK8560274.1	Exocyst complex component (SEC6). Vesicle trafficking, exocytic trafficking	Exocyst complex component sec	Exocyst complex component SEC6
	SNP035419- SNP035415^*^	6	408,765,457–408,765,593	*g24166.t1*	CAK8571430.1	AGC-VIIIa/plant-AGC1 protein kinase (AGC1), transferring phosphorus-containing group	Protein kinase domain containing	Serine/threonine protein kinase KIPK
	SNP035962	6	478,197,292	*g24512.t1*	CAK8571811.1	Inosine and uridine kinase (PNK) of purine salvage pathway. Nucleotide metabolism	PfkB domain containing protein	Unnamed protein product
RS	SNP000794	1	595,681,698	*g3062.t1*	CAK8533241.1	Not classified	O-acyltransferase wax ester synthase/diacylglycerol acyltransferase protein	Diacylglycerol acyltransferase
	SNP032149	5	561,173,187	*g21044.t1*	CAK8567981.1	Not classified	Glutamyl-tRNA(Gln) amidotransferase A subunit	Fatty-acid amide hydrolase
	SNP041226	7	531,394,642	*g29459.t1*	CAK8577011.1	Callose synthase. Cell wall organisation	1,3-beta glucan synthase	Callose synthase
FS and RS	SNP007517	6	525,489,889	*g24922.t1*	CAK8572265.1	Ethylene receptor protein. Phytohormone action, ethylene perception and signal transduction	Ethylene receptor	Protein EIN4. Ethylene receptor

^*^Markers in linkage disequilibrium

## Discussion

This study characterised the response of a diverse global grass pea collection to *A.* *euteiches*, confirming grass pea susceptibility to this pathogen and identifying two resistant and 10 partially resistant accessions in this crop. The broad range of FS and RS observed in the collection presented a suitable base, together with an improved acquired SNP data set, to study the genetic architecture behind this response. The genome-wide association study identified 20 significantly associated markers linked to resistance across five of the seven grass pea chromosomes, confirming its polygenic nature and allowing the proposal of potential candidate genes involved in the response. To our knowledge, this is the first application of association analysis to explore grass pea resistance to *A.* *euteiches*.

A wide range in FS and RS was observed among the 169 evaluated grass pea accessions, confirming the susceptibility of this crop to *A.* *euteiches*. Nevertheless, when comparing RS severity with results from similar pea infection studies, grass pea appears to be, on average, less susceptible than pea, with most of the evaluated accessions classified as partially susceptible in both of the evaluated symptoms. Also, as previously described in pea, a significant moderate correlation was observed between FS and RS (Rodriguez-Mena et al. 2024). In contrast to pea, this study identified two accessions with very high levels of resistance, a situation that has never been described in pea, where only partial resistance has been described (Desgroux et al. 2016; McGee et al. 2012; Rodriguez-Mena et al. 2024). Notably, grass pea accession PI283570_A and accession PI283580 exhibited complete resistance to the tested isolate, supporting the potential existence of intra-specific genetic variability in response to *A.* *euteiches* infection, as previously reported in faba bean (Leprévost et al. 2026), or the existence of race-resistant accessions in alfalfa (Giles et al. 2022). Otherwise, the pea origin of the tested isolate caused a non-host-specific and less aggressive infection (Moussart et al. 2024); the identification of grass pea-specific isolates could further validate these results. Additionally, ten accessions showed partial resistance. One of these partially resistant accessions is STUDENICA, a commercial variety with desirable agronomic traits that presents a promising candidate for cultivation in soils infested with *A.* *euteiches* (Gonçalves et al. 2024). Among the tested resistant accessions, PI283580, BGE17185 and PTLS1006 also demonstrated low symptom levels in response to another soil-borne pathogen, *Fusarium* *oxysporum* f. sp. *pisi* (Sampaio et al. 2021). Given the high frequency of co-infection of *A.* *euteiches* and *Fusarium* spp. in pea, accessions with resistance to both pathogens represent valuable resources for breeding broadly resistant cultivars (Sharma et al. 2022; Trenk et al. 2024). Regarding seed characteristics, light-coloured, large seed accessions showed lower FS and RS compared to dark, small seed accessions. This aligns with the lower RS observed in Mediterranean seeds, which are typically larger and lighter, compared to the predominantly small, dark seeds of South Asian origin. The same characteristics were associated with more resistant accessions to *Fusarium* *oxysporum* f. sp. *pisi* in the evaluated collection that could indicate similar mechanisms behind grass pea resistance to these two soil-borne pathogens (Sampaio et al. 2021).

The broad phenotypic variation among the evaluated grass pea after *A.* *euteiches* inoculation, together with the high heritability associated with both foliar and root symptoms, supports the suitability of this collection to perform genetic studies. The performed GWAS identified 20 significant molecular markers, 12 associated with FS, 7 with RS, and one common marker between both symptoms. Among these, two markers, SNP032149 and the common marker between symptoms SNP007517, were consistently detected by two models (BLINK and FarmCPU), reinforcing their potential relevance to Aphanomyces root rot resistance in grass pea. Clear allelic differences between resistant and susceptible groups were observed for the associated markers. These differences were more pronounced for foliar-related SNPs than for root-related ones, likely due to a higher incidence of missing/heterozygotes in accessions classified as resistant based on RS.

The significant markers identified in this study were distributed across five of the seven grass pea chromosomes. This pattern, together with the moderate proportion of PVE per marker, supports the notion that complex polygenic resistance to *A.* *euteiches* is present in grass pea, as previously reported in other legumes (Leprévost et al. 2026). However, the resistance nature of the two described resistant accessions requires further investigation to determine whether they exhibit monogenic or polygenic resistance. Given the high genetic diversity in *A.* *euteiches* populations and its hemibiotrophic lifestyle, polygenic quantitative resistance may provide a more durable long-term management strategy than monogenic based resistance (Mundt 2014; Kälin et al. 2022). The GWAS highlighted six regions, two on chromosome 5 and four on chromosome 6, that harbour closely located significantly associated markers, suggesting the presence of potential quantitative trait loci associated with Aphanomyces root rot resistance in grass pea. The chromosome 5 regions, spanned from 193,375,403 (SNP005290) to 241,052,867 (SNP005347) and from 508,589,669 (SNP031472) to 561,173,187 (SNP032149). The four regions of closely located markers in chromosome 6 were from 395,296,031 bp (SNP035346) to 408,765,593 bp (SNP035419), a second from 478,139,587 bp (SNP035948) to 478,197,292 bp (SNP035962), a third from 525,489,889 bp (SNP007517) to 525,490,364 bp (SNP007520) and the last from 616,109,525 bp (SNP038080) to 616,109,553 bp (SNP038082). The chromosomal distribution of associated markers differed from those in other susceptible species (pea, lentil, faba bean or barrel medic), lacking the identification of a conserved genomic pattern with previous studies (Leprévost et al. 2026). Further investigation is required to confirm the roles of these candidate loci.

Examining the genomic location of the significantly associated markers led to the identification of 7 candidate genes potentially involved in *A.* *euteiches* resistance in grass pea. These genes are predicted to participate in various biological processes, including vesicle trafficking (*g14004.t1*), signal transduction (*g24166.t1*), nucleotide metabolism (*g24512.t1*), fatty-acid metabolism (*g3062.t1* and *g21044.t1*), cell wall synthesis (*g29459.t1*) and ethylene signalling (*g24922.t1*). Notably, serine/threonine protein kinases (*g24166.t1*) and ethylene-related proteins (*g24922.t1*) have previously been associated with *A.* *euteiches* response in other legumes (Nyamsuren et al. 2003; Afzal et al. 2008; Badis et al. 2015; Desgroux et al. 2016; Jacquet and Bonhomme 2019; Rodriguez-Mena et al. 2025; Pandit et al. 2025). Serine/threonine protein kinases are involved in signalling and plant defences, while the ethylene pathway leads to lignin accumulation (Djébali et al. 2011; Desgroux et al. 2016). Lignin accumulation enables the formation of a protective ring around the central cylinder in infected roots, restricting pathogen entry and preserving root function (Djébali et al. 2011). Regarding the other candidate genes, evidence from non-Aphanomyces root rot studies suggests that they may also play defence-related roles against *A.* *euteiches*. For instance, infection increases the presence of several fatty acids (*g3062.t1* and *g21044.t1*) in pea which has been proposed to mediate reactive oxygen species (ROS) production, contributing to *R* gene–mediated resistance (Hossain et al. 2024). Similarly, a gene involved in callose synthesis (*g29459.t1*) may play a role in reinforcing cell walls at infection sites, as callose and phenolic compound deposition have been shown to limit fungal hyphal growth at the infection point (German et al. 2022). Although components of the exocyst complex (*g14004.t1*) have been associated with plant immunity, their specific role in *A.* *euteiches* resistance remains poorly understood (De la Concepcion 2023). Likewise, the contribution of the in silico detected genes required further investigation. *In* *planta* validation of these genes could clarify their role in the resistance and contribute to characterise the mechanism behind grass pea response to *A.* *euteiches*.

Phenotypic evaluation of grass pea under field conditions is required to assess the potential impact of *A.* *euteiches* in natural environments and to validate the identified markers and associated candidate genes. Evaluating additional *A.* *euteiches* isolates would also clarify whether the observed susceptibility in grass pea is specific to RB84 or applicable to other isolates. Such analysis would also reveal whether the genomic regions identified in this study have a major or minor effect depending on the tested pathotype, as occurs in pea (Leprévost et al. 2026). The RB84 isolate was selected in this study due to its documented capacity to infect several legume species, confirming also in this work its ability to infect grass pea (Leprévost et al. 2026). However, it is a pea isolate, which implies that the characterised response is non-host specific. Should grass pea isolates exist, they might elicit a stronger response and potentially overcome the complete resistance identified in this study.

Altogether, this study confirmed a compatible interaction between grass pea and *A.* *euteiches* RB84 isolate, but with variable levels of polygenic resistance. The tested collection exhibited a wide range of foliar and root symptomatology after inoculation, allowing the identification of resistant and partially resistant accessions, which can be exploited in future breeding programmes. The performed GWAS allowed the identification of significant associated SNPs on five of the seven grass pea chromosomes, explaining only a percentage of the observed variability, consistent with the quantitative nature of resistance to *A.* *euteiches* described in other legumes. A notable concentration of significant markers on chromosomes 5 and 6, suggests the presence of resistance-related loci in these regions. Additionally, the significant associated markers enabled the identification of seven candidate genes potentially involved in the grass pea response to *A.* *euteiches*. Among them, genes encoding serine/threonine protein kinases and ethylene receptors are noteworthy due to their previously reported roles in *A.* *euteiches* defence in other legume species. Nevertheless, functional validation is required to clarify their role in the response and to guide their use in precision breeding approaches. These might also be potentially valuable for pea breeding if crossability barriers are overcome. Therefore, this study opens new avenues to study grass pea response against *A.* *euteiches* as well as proposed resistance-associated loci and candidate genes involved in the response.

## Supplementary Information

Below is the link to the electronic supplementary material.

Supplementary file1 (XLSX 8053 KB)

Supplementary file2 (PDF 468 KB)

## Data Availability

All data sets generated in this study are provided in the article supplementary files (Online resources).

## References

[CR1] Afzal AJ, Wood AJ, Lightfoot DA (2008) Plant receptor-like serine threonine kinases: roles in signaling and plant defense. Mol Plant Microbe Interact 21(5):507–517. 10.1094/MPMI-21-5-0507 18393610

[CR2] Aphalo PJ, Slowikowski K, Mouksassi S (2024) ggmisc: Miscellaneous Extensions to “ggplot2”. R package version 0.6.2.9001. https://docs.r4photobiology.info/ggpmisc/

[CR3] Astle W, Balding DJ (2009) Population structure and cryptic relatedness in genetic association studies. Statist Sci 24(4):451–471. 10.1214/09-STS307

[CR4] Badis Y, Bonhomme M, Lafitte C, Huguet S, Balzergue S, Dumas B, Jacquet C (2015) Transcriptome analysis highlights preformed defences and signalling pathways controlled by the *prAe1* quantitative trait locus (QTL), conferring partial resistance to *Aphanomyces**euteiches* in *Medicago**truncatula*. Mol Plant Pathol 16:973–986. 10.1111/mpp.12253 PMC6638387 25765337

[CR5] Becking T, Kiselev A, Rossi V, Street-Jones D, Grandjean F, Gaulin E (2022) Pathogenicity of animal and plant parasitic *Aphanomyces* spp. and their economic impact on aquaculture and agriculture. Fungal Biol Rev 40:1–18. 10.1016/j.fbr.2021.08.001

[CR6] Bonhomme M, Fariello MI, Navier H, Hajri A, Badis Y, Miteul H, Samac DA, Dumas B, Baranger A, Jacquet C, Pilet-Nayel M-L (2019) A local score approach improves GWAS resolution and detects minor QTL: application to *Medicago**truncatula* quantitative disease resistance to multiple *Aphanomyces**euteiches* isolates. Heredity 123:517–531. 10.1038/s41437-019-0235-x PMC6781128 31138867

[CR7] Chao J, Li Z, Sun Y, Aluko OO, Wu X, Wang Q, Liu G (2021) MG2C: a user-friendly online tool for drawing genetic maps. Mol Hortic 1:16. 10.1186/s43897-021-00020-x PMC10514940 37789491

[CR8] Chatterton S, Schwinghamer TD, Pagé A, Davidson RB, Harding MW, Banniza S (2023) Inoculum dose–disease response relationships for the pea root rot pathogen*,**Aphanomyces**euteiches*, are dependent on soil type and other pathogens. Front Plant Sci 14:1115420. 10.3389/fpls.2023.1115420 PMC10205554 37235016

[CR9] Chen Z, Boehnke M, Wen X, Mukherjee B (2021) Revisiting the genome-wide significance threshold for common variant GWAS. G3 (Bethesda) 11(2):jkaa056. 10.1093/g3journal/jkaa056 PMC8022962 33585870

[CR10] Christensen RHB (2019) Ordinal: regression models for ordinal data. R package version 2023.12–4.1. https://CRAN.R-project.org/package=ordinal.

[CR11] De la Concepcion JC (2023) The exocyst complex is an evolutionary battleground in plant-microbe interactions. Curr Opin Plant Biol 76:102482. 10.1016/j.pbi.2023.102482 37924562

[CR12] Desgroux A, L’Anthoëne V, Roux-Duparque M, Rivière JP, Aubert G, Tayeh N, Moussart A, Mangin P, Vetel P, Piriou C, McGee RJ, Coyne CJ, Burstin J, Baranger A, Manzanares-Dauleux M, Bourion V, Pilet-Nayel M-L (2016) Genome-wide association mapping of partial resistance to *Aphanomyces**euteiches* in pea. BMC Genomics 17:124. 10.1186/s12864-016-2429-4 PMC4761183 26897486

[CR13] Ditzler L, van Apeldoorn DF, Pellegrini F, Antichi D, Bàrberi P, Rossing WAH (2021) Current research on the ecosystem service potential of legume inclusive cropping systems in Europe. A review. Agron Sustain Dev 41:26. 10.1007/s13593-021-00678-z

[CR14] Djébali N, Mhadhbi H, Lafitte C, Dumas B, Esquerré-Tugayé MT, Aouani ME, Jacquet C (2011) Hydrogen peroxide scavenging mechanisms are components of *Medicago**truncatula* partial resistance to *Aphanomyces**euteiches*. Eur J Plant Pathol 131:559–571. 10.1007/s10658-011-9831-1

[CR15] Estaghvirou SBO, Ogutu JO, Piepho HP (2014) Influence of outliers on accuracy estimation in genomic prediction in plant breeding. G3 (Bethesda) 4:2317–2328. 10.1534/g3.114.011957 PMC4267928 25273862

[CR16] Gaulin E, Jacquet C, Bottin A, Dumas B (2007) Root rot disease of legumes caused by *Aphanomyces**euteiches*. Mol Plant Pathol 8:539–548. 10.1111/j.1364-3703.2007.00413.x 20507520

[CR17] German L, Yeshvekar R, Benitez-Alfonso Y (2022) Callose metabolism and the regulation of cell walls and plasmodesmata during plant mutualistic and pathogenic interactions. Plant Cell Environ 46:391–404. 10.1111/pce.14510 PMC10107507 36478232

[CR18] Giles JM, Tordsen CL, Rebstock TR, Bucciarelli B, Samac DA, Sathoff AE (2022) Management strategies and distribution of *Aphanomyces* root rot of alfalfa (*Medicago**sativa*), a continuing threat to forage production in the United States. Plant Pathol 71:1231–1240. 10.1111/ppa.13563

[CR19] Glaubitz JC, Casstevens TM, Lu F, Harriman J, Elshire RJ, Sun Q, Buckler ES (2014) TASSEL-GBS: a high capacity genotyping by sequencing analysis pipeline. PLoS ONE 9(2):e90346. 10.1371/journal.pone.0090346 PMC3938676 24587335

[CR20] Gonçalves L, Rubiales D, Bronze MR, Vaz Patto MC (2022) Grass pea (*Lathyrus**sativus* L.)—a sustainable and resilient answer to climate challenges. Agronomy (Basel) 12(6):1324. 10.3390/agronomy12061324

[CR21] Gonçalves L, Rubiales D, Lourenço M, Vaz Patto MC (2024) Exploring grass pea (*Lathyrus sativus* L.) genetic diversity in Mediterranean changing climate conditions. Eur J Agron 156:127142. 10.1016/j.eja.2024.127142

[CR22] Hoagland DR, Arnon DI (1938) The water-culture method for growing plants without soil. Circ Calif Agric Exp Stn 347:39

[CR23] Hossain S, Bergkvist G, Berglund K, Mårtensson A, Persson P (2012) *Aphanomyces* pea root rot disease and control with special reference to impact of brassicaceae cover crops. Acta Agric Scand Sec B Soil Plant Sci 62:477–487. 10.1080/09064710.2012.668218

[CR24] Hossain Z, Zhao S, Luo X, Liu K, Li L, Hubbard M (2024) Deciphering *Aphanomyces**euteiches*-pea-biocontrol bacterium interactions through untargeted metabolomics. Sci Rep 14:8877. 10.1038/s41598-024-52949-w PMC11024177 38632368

[CR25] Hughes TJ, Grau CR (2007) Aphanomyces root rot or common root rot of legumes. Plant Health Instr. 10.1094/PHI-I-2007-0418-01

[CR26] Jacquet C, Bonhomme M (2019) Deciphering resistance mechanisms to the root rot disease of legumes caused *by**Aphanomyces**euteiches* with *Medicago**truncatula* genetic and genomic resources. In: de Bruijn F (ed) The model legume *Medicago**truncatula*. Wiley, New York, pp 307–316

[CR27] Kälin C, Berlin A, Kolodinska Brantestam A-K, Dubey M, Arvidsson AK, Riesinger P, Elfstrand M, Karlsson M (2022) Genetic diversity of the pea root pathogen *Aphanomyces**euteiches* in Europe. Plant Pathol 71:1570–1578. 10.1111/ppa.13598

[CR28] Kilian A, Wenzl P, Huttner E, Carling J, Xia L, Blois H, Caig V, Heller-Uszynska K, Jaccoud D, Hopper C, Aschenbrenner-Kilian M, Evers M, Peng K, Cayla C, Hok P, Uszynski G (2012) Diversity arrays technology: a generic genome profiling technology on open platforms. Methods Mol Biol 888:67–89. 10.1007/978-1-61779-870-2_5 22665276

[CR29] Leitão ST, Rubiales D, Vaz Patto MC (2023) Identification of novel sources of partial and incomplete hypersensitive resistance to rust and associated genomic regions in common bean. BMC Plant Biol 23:610. 10.1186/s12870-023-04619-8 PMC10691055 38041043

[CR30] Lenth R (2025) Estimated marginal means, aka least-squares means. R package version 1.11.2–80001, https://rvlenth.github.io/emmeans/

[CR31] Leprévost T, Boutet G, Lesné A, Rivière J-P, Vetel P, Glory I, Miteul H, Le Rat A, Dufour P, Regnault-Kraut C, Sugio A, Lavaud C, Pilet-Nayel M-L (2023) Advanced backcross QTL analysis and comparative mapping with RIL QTL studies and GWAS provide an overview of QTL and marker haplotype diversity for resistance to Aphanomyces root rot in pea (*Pisum**sativum*). Front Plant Sci 14:1189289. 10.3389/fpls.2023.1189289 PMC10569610 37841625

[CR32] Leprévost T, Imbert B, Boutet G, Lavaud C, Miteul H, Leduc A, Aubert G, Kreplak J, Carrillo-Perdomo E, Uhdre R, Sari H, Bourland B, Caron CT, Tayeh N, Ma Y, Coyne CJ, Sugio A, Pilet-Nayel M-L (2026) Comparative genomic analysis of QTL for resistance to *Aphanomyces**euteiches* between pea, lentil, faba bean and the model species *Medicago**truncatula*. Theor Appl Genet 139:2. 10.1007/s00122-025-05095-4 41369777

[CR33] Li H, Handsaker B, Wysoker A, Fennell T, Ruan J, Homer N, Marth G, Abecasis G, Durbin R (2009) The sequence alignment/map format and SAMtools. Bioinformatics 25(16):2078–2079. 10.1093/bioinformatics/btp352 PMC2723002 19505943

[CR34] Li H (2013) Aligning sequence reads, clone sequences and assembly contigs with BWA-MEM. ArXiv.

[CR35] McGee RJ, Coyne CJ, Pilet-Nayel ML, Moussart A, Tivoli B, Baranger A, Hamon C, Vandemark G, McPhee K (2012) Registration of pea germplasm lines partially resistant to Aphanomyces root rot for breeding fresh or freezer pea and dry pea types. J Plant Regist 6(2):203–207. 10.3198/jpr2011.03.0139crg

[CR36] Melo ATO, Bartaula R, Hale I (2016) GBS-SNP-CROP: a reference-optional pipeline for SNP discovery and plant germplasm characterization using variable length, paired-end genotyping-by-sequencing data. BMC Bioinform 17(1):29. 10.1186/s12859-016-0879-y PMC4709900 26754002

[CR37] Moussart A, Lavaud C, Onfroy C, Leprévost T, Pilet-Nayel M-L, Le May C (2024) Pathotype characterization of *Aphanomyces**euteiches* isolates collected from pea breeding nurseries. Front Plant Sci 15:1332976. 10.3389/fpls.2024.1332976 PMC11007135 38606076

[CR38] Mundt CC (2014) Durable resistance: a key to sustainable management of pathogens and pests. Infect Genet Evol 27:446–455. 10.1016/j.meegid.2014.01.011 PMC4117828 24486735

[CR39] Nakagawa S, Schielzeth H (2010) Repeatability for Gaussian and non-Gaussian data: a practical guide for biologists. Biol Rev Camb Philos Soc 85:935–956. 10.1111/j.1469-185X.2010.00141.x 20569253

[CR40] Nyamsuren O, Colditz F, Rosendahl S, Tamasloukht MB, Bekel T, Meyer F, Kuester H, Franken P, Krajinski F (2003) Transcriptional profiling of *Medicago**truncatula* roots after infection with *Aphanomyces**euteiches* (oomycota) identifies novel genes upregulated during this pathogenic interaction. Physiol Mol Plant Pathol 63:17–26. 10.1016/j.pmpp.2003.09.001

[CR41] Osuna‐Caballero S, Rubiales D, Rispail N (2024) Genome‐wide association study uncovers pea candidate genes and metabolic pathways involved in rust resistance. Plant Genome 17(4):e20510. 10.1002/tpg2.20510 PMC11628884 39472763

[CR42] Pandit S, Halane H, Singer SD, Zhin-Loong Lim N, Dhaubhadel S, Goyal R, Zhang W, Foroud NA, Schultz E, Chatterton S (2025) Transcriptomic and targeted metabolomic analyses of partially resistant and susceptible pea genotypes reveal differential defense responses during their interaction with *Aphanomyces**euteiches*. Legume Sci 7:e70067. 10.1002/leg3.70067

[CR43] Papaïx J, Goyeau H, Du Cheyron P, Monod H, Lannou C (2011) Influence of cultivated landscape composition on variety resistance: an assessment based on wheat leaf rust epidemics. New Phytol 191:1095–1107. 10.1111/j.1469-8137.2011.03764.x 21585391

[CR44] Papavizas GC, Ayers WA (1974) *Aphanomyces* species and their root diseases in pea and sugarbeet: a review. Technical bulletins No. 1485, United States Department of Agriculture, Economic Research Service.

[CR45] Pilet-Nayel M-L, Moury B, Caffier V, Montarry J, Kerlan M-C, Fournet S, Durel C-E, Delourme R (2017) Quantitative resistance to plant pathogens in pyramiding strategies for durable crop protection. Front Plant Sci 8:1838. 10.3389/fpls.2017.01838 PMC5664368 29163575

[CR46] R Core Team (2024) R: a language and environment for statistical computing. https://www.R-project.org/

[CR47] Resende RT, Resende MDV, Silva FF, Azevedo CF, Takahashi EK, Silva-Junior OB, Grattapaglia D (2017) Regional heritability mapping and genome-wide association identify loci for complex growth, wood and disease resistance traits in *Eucalyptus*. New Phytol 213:1287–1300. 10.1111/nph.14266 28079935

[CR48] Rodriguez-Mena S, Rubiales D, González M (2024) Identification of sources of resistance to *Aphanomyces* root rot in *Pisum*. Plants 13(17):2454. 10.3390/plants13172454 PMC11397196 39273939

[CR49] Rodriguez-Mena S, Vaz Patto MC, Leitão ST, Rubiales D, González M (2025) Identification of genomic regions associated with partial resistance to Aphanomyces root rot in pea. Plant Genome 18:e70164. 10.1002/tpg2.70164 PMC12666727 41321282

[CR50] Sampaio AM, Alves ML, Pereira P, Valiollahi E, Santos C, Šatović Z, Rubiales D, de Sousa Araújo S, van Eeuwijk F, Vaz Patto MC (2021) Grass pea natural variation reveals oligogenic resistance to *Fusarium oxysporum* f. sp. *pisi*. Plant Genome 14(3):e20154. 10.1002/tpg2.20154 PMC12807200 34617677

[CR51] Schwacke R, Ponce-Soto GY, Krause K, Bolger AM, Arsova B, Hallab A, Gruden K, Stitt M, Bolger ME, Usadel B (2019) MapMan4: a refined protein classification and annotation framework applicable to multi-omics data analysis. Mol Plant 12(6):879–892. 10.1016/j.molp.2019.01.003 30639314

[CR52] Sharma A, Rani M, Lata H, Thakur A, Sharma P, Kumar P, Jayswal DK, Rana RS (2022) Global dimension of root rot complex in garden pea: current status and breeding prospective. Crop Prot 158:106004. 10.1016/j.cropro.2022.106004

[CR53] Solovieva I, Miteva-Bölter P, Knez M, Bessai A-K, Barilli E, Kasperczyk N, Ranic M, Gurinovic M, Luna Casado PJ, Alba Morales N et al (2025) Exploring the potential and challenges of *Lathyrus**sativus* (grass pea) in European agri-food value chains: a cross-country analysis. Sustainability 17(8):3283. 10.3390/su17083283

[CR54] Storey JD, Tibshirani R (2003) Statistical significance for genome wide studies. Proc Natl Acad Sci U S A 100(16):9440–9445. 10.1073/pnas.1530509100 PMC170937 12883005

[CR55] Trenk NK, Pacheco-Moreno A, Arora S (2024) Understanding the root of the problem for tackling pea root rot disease. Front Microbiol 15:1441814. 10.3389/fmicb.2024.1441814 PMC11540676 39512933

[CR56] Tripodi P (2022) Development, preparation, and curation of high-throughput phenotypic data for genome-wide association studies: a sample pipeline in R. Methods Mol Biol 2481:105–125. 10.1007/978-1-0716-2237-7_7 35641761

[CR57] Vigouroux M, Novák P, Oliveira LC, Santos C, Cheema J, Wouters RHM, Paajanen P, Vickers M, Koblížková A, Vaz Patto MC, Macas J, Steuernagel B, Martin C, Emmrich PMF (2024) A chromosome-scale reference genome of grasspea (*Lathyrus**sativus*). Sci Data 11:1035. 10.1038/s41597-024-03868-y PMC11437036 39333203

[CR66] VSN International: Genstat for Windows 24th Edition. Hemel Hempstead, UK: VSN International; 2022 Web page: http://genstat.co.uk/

[CR58] Wang J, Zhang Z (2021) GAPIT version 3: boosting power and accuracy for genomic association and prediction. Genom Proteom Bioinform 19(4):629–640. 10.1016/j.gpb.2021.08.005 PMC9121400 34492338

[CR59] Wang Q (2016). QQperm: permutation based QQ plot and inflation factor estimation. R package version 1.0.1, https://cran.r-project.org/web/packages/QQperm

[CR60] Wickham H (2016) ggplot2: elegant graphics for data analysis. Springer-Verlag, New York

[CR61] Wikström J, Chaudhary S, Persson L, Wikström M, Fatehi J, Karlsson M (2025) Distribution of plant pathogenic *Aphanomyces* species in Sweden, Denmark and Lithuania and its relationship with soil factors. J Plant Pathol. 10.1007/s42161-025-01835-z

[CR62] Willsey T, Patey J, Vucurevich C, Chatterton S, Carcamo H (2021) Evaluation of foliar and seed treatments for integrated management of root rot and pea leaf weevil in field pea and faba bean. Crop Prot 143:105538. 10.1016/j.cropro.2021.105538

[CR63] Wu L, Chang K-F, Conner RL, Strelkov S, Fredua-Agyeman R, Hwang S-F, Feindel D (2018) *Aphanomyces**euteiches*: a threat to Canadian field pea production. Engineering 4(4):542–551. 10.1016/j.eng.2018.07.006

[CR64] Wu L, Fredua-Agyeman R, Hwang S-F, Chang K-F, Conner RL, McLaren DL, Strelkov SE (2021) Mapping QTL associated with partial resistance to *Aphanomyces* root rot in pea (*Pisum**sativum* L.) using a 13.2 K SNP array and SSR markers. Theor Appl Genet 134:2965–2990. 10.1007/s00122-021-03871-6 34129066

[CR65] Zhang Z (2023) User manual for GAPIT. Genomic association and prediction integrated tool version 3

