# Supplementary material for: Aphanomyces euteiches causes disease in Lathyrus sativus with a globally diverse, polygenic resistance landscape
Source: Theor Appl Genet. 2026 Sep 21;139(10):272. doi: 10.1007/s00122-026-05372-w (PMC13593742; doi:10.1007/s00122-026-05372-w)
Supplement: Supplementary file 1 [file 122_2026_5372_MOESM2_ESM.pdf]

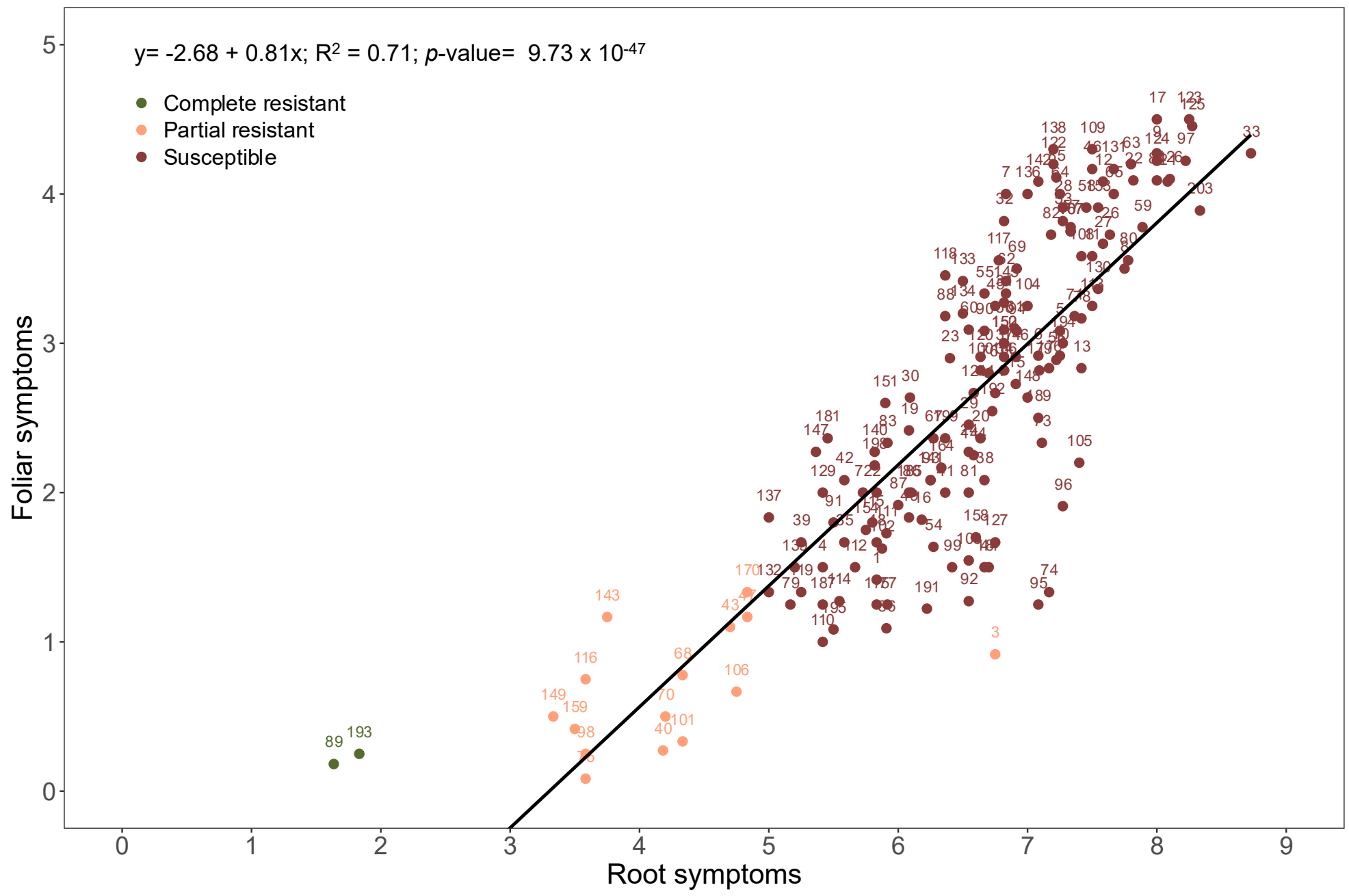

Fig. S1 Average foliar (FS) and root symptoms (RS) of the 169 grass pea evaluated accessions ID labelled under controlled conditions 20 days after *Aphanomyces euteiches* inoculation. Accessions are coloured based on their classification as complete resistance (FS < 0.5 and RS < 2), partial resistance ($0.5 \leq$ FS < 1 and $2 \leq$ RS < 5) and susceptible (FS $\geq$ 1 and RS $\geq$ 5). Equation of the linear adjustment, correlation coefficient and *p*-value are included within the graph. Means were calculated using the evaluations of four biological replicates across the three temporal rounds (12 plants per accession).

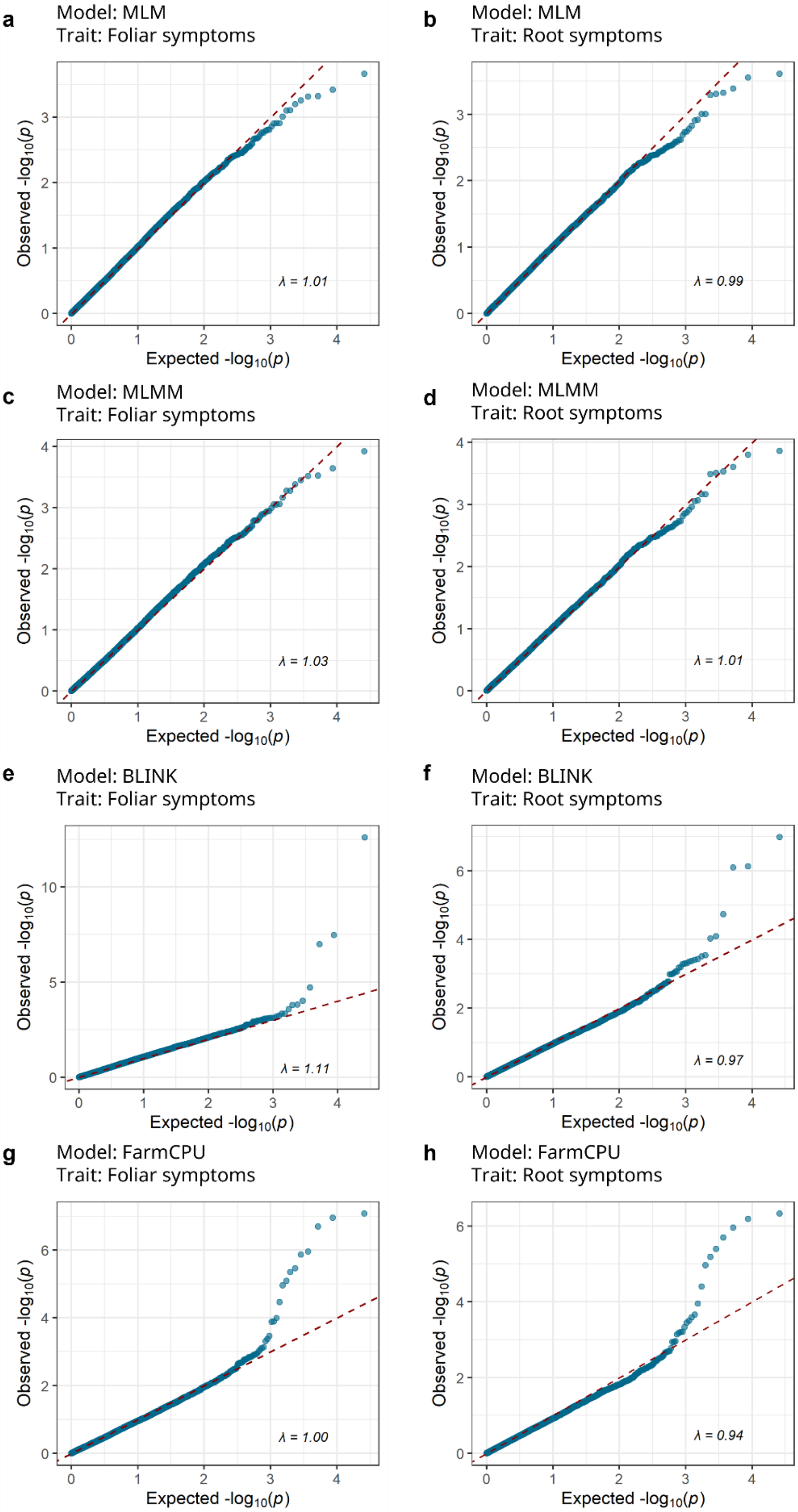

Fig. S2 Quantile-Quantile (QQ) plots from the association mapping for foliar (left column) and root (right column) symptoms using single locus mixed linear model (MLM) (a -b), multiple locus mixed linear model (MLMM) (c-d), multi-locus Bayesian information and linkage disequilibrium iteratively nested keyway (BLINK) (e-f), and Fixed and random model circulating probability unification (FarmCPU) (g-h) models.

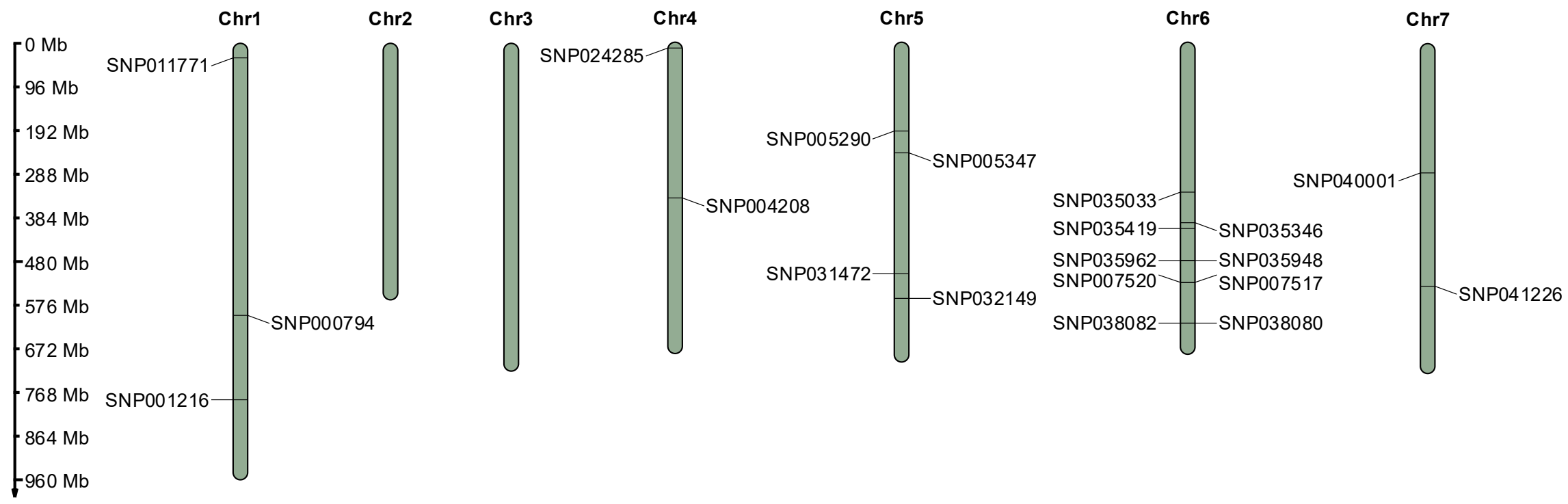

Fig. S3. Distribution of the 20 markers most significantly associated with *A. euteiches* infection ratings along the seven grass pea chromosomes.
